# Intraoral Vestibular Pressure: Quantification and Implications on Gum Trophism

**DOI:** 10.1155/ijod/8857779

**Published:** 2026-01-30

**Authors:** Davide Farronato, Gabriele Dani, Piero Antonio Zecca, Andrea Moriondo, Leonardo Romano, Lorenzo Azzi

**Affiliations:** ^1^ Departement of Medicine and Surgery, University of Insubria, Varese, Lombardy, Italy, uninsubria.eu

**Keywords:** intraoral pressure, mechanotransduction, microcirculation, mucosa growth, peri-implant mucosa, suction chamber

## Abstract

**Introduction:**

This pioneering study delves into the dynamics of intraoral pressure, examining its consistency across different subjects and conditions. The null hypothesis is a different pressure at rest between the intraoral zone and the atmospheric pressure.

**Materials and Methods:**

Utilizing a novel apparatus and method, based on the concept of communicating vessels, the research aimed to measure resting intraoral pressure at the vestibular level.

**Results:**

The findings revealed no significant variations in intraoral pressure across different individuals or under varying sectors of analysis or wakefulness versus sleep state. The average resting pressure in the oral vestibule was identified to be −1.25 mmHg, suggesting a residual relative negative pressure post‐swallowing that could aid in various physiological functions.

**Discussion:**

The values found align with previous studies on swallowing and with studies that have attempted to measure the intraoral pressure exerted by the muscles of cheeks and lips. The study further supports existing theories on peri‐implant tissue maturation and the role of oral vacuum in promoting gingival growth. Specifically, it suggests a potential impact of the negative pressure gradient on gingival microcirculation as an explanation for the volumetric growth of gingival tissue.

**Conclusion:**

Within the limitation of the present sample size, this research confirms the hypothesis related to intraoral pressure in understanding various physiological processes within the oral cavity, paving the way for further exploration in this domain with implications for dental and oral health practices.

## 1. Introduction

The mechanism by which the growth of soft tissues around implants occurs has been debated for many years, but, despite a convergence of clinical observations, the fundamental reason supporting these findings with evidence still remains unclear. The design of the emergence profile is shown to be critical for tissue maturation and shaping [1] and, nevertheless, strongly correlated to anatomical [2] and biomechanical factors [3]. The esthetic biological contour concept [4] explains the importance of a narrow and concave subcritical contour and a convex critical zone.

Recently, a retrospective clinical study observed the behavior of the mucosa around implant‐supported prostheses [5] with the above‐described emergence profile design.

According to Agabiti, the prosthodontic outline, once emerged from the free gingival line, offers a sort of protection to the gum through a convex shape that supports the muscles of cheeks and lips, generating an empty space that, within biological limits, could be filled with the gingiva. The researchers suggested that this phenomenon may be due to a negative pressure gradient within the oral cavity that, through suction, promotes the growth of peri‐implant mucosa [5].

In the context of the Biologically Oriented Preparation Technique [6], several studies have been conducted on gum healing following dental preparation. It has been histologically observed that the extracellular matrix undergoes mechanical stresses, including oral vacuum [7]. These mechanical stresses are transmitted to the cell cytoskeleton through a mechanotransduction mechanism leading to gingival tissue growth [7]. The oral vacuum that forms during swallowing has been confirmed and measured by various studies on swallowing through the realization of different devices. First, the group led by Petra Santander [8] used a silicon shield that covers the vestibular part of the dental arches, linked to a silicon tube making a ring on the upper part of the tongue. Lastly, two holes are made: one to consent water aspiration from an extra‐oral syringe or food injection, and the second to measure the pressure under the palate with a sensor (GMSD2BR) in a range between −1.000 and 2.000 mbar, resolution 1 mbar, and frequency 1 kHz. It is linked with a computerized pressure gauge (GDUSB 1000). Three swallowing conditions were measured: an active bolus intake (ABI) of water, a passive bolus application of a water bolus (PWA), and a passive application of a gel bolus (PGA). Negative pressures were registered with a median value of −278.9 mbar during active water‐bolus intake, of −24.2 mbar during passive water‐bolus intake, and of −29.4 mbar during passive gel‐bolus intake, showing significant differences in pressure values depending on the bolus consistency and application [8]. Kieser [9], instead, developed a cobalt‐chrome palatal device with a 0.3–0.5 thickness labial arch to measure intraoral pressure changes in different parts of the oral cavity. To this structure, eight miniaturized pressure transducers were fixed (105S type, *Precision Measurement Company*, Michigan, USA). After swallowing 10 mL of water in an upright position on the labial side, values of the first molar followed a negative pattern, peaking at −15 kPa. The labial side of the canine passed rapidly from 10 to 33 kPa. While on the palatal part, it appears to be similar to the first molar, but the latter fell to −13 kPa before rising to 9 kPa; the canine pressure instead rapidly increased to 22 kPa before returning to its resting level of 4 kPa. About the incisor, on the labial part, the pattern is similar to the one of the first molar except for the final peak of −12 kPa. On the other hand, on the palatal side, the pressure variation profile was different, starting from −20 kPa at the beginning of swallowing, then slowly rising to 10 kPa and returning to a rest level of 4 kPa after another peak of 10 kPa [9]. Engelke et al. [10] then realized a pressure measurement with one digital pressure gauge in the vestibular inter‐occlusal space and one in the palatal area. The device consisted of an intravenous polyethylene catheter with a standardized extraction hood at the end tip. Both sensors were placed on a PVC flexible tube linked to a pressure detector (GMSD 350 MR) capable of detecting variations in a range of 500 mbar (+ 100 to −400 mbar) and 0.1 mbar of resolution. The results were significantly different in the two areas. Suggesting they are different functions, for example, the tongue creates a seal with the palate where the suction effect gives the possibility to release elevator muscles of the mandible. In clinical research [10], the pressure within the space between teeth was assessed. After swallowing, there was a constantly maintained average relative pressure of −48.79 mbar in the inter‐occlusal compartment, which persisted with closed lips until the subsequent opening. These studies confirm the presence of an intraoral pressure lower than the atmospheric one during swallowing. However, no study has evaluated the resting vestibular intraoral pressure. The intention is to study the resting condition rather than active phases of chewing or swallowing because the forces present at rest, as they act over time, have the greatest impact. Several authors in the literature have attempted to measure intraoral pressure in various ways and for different purposes. Some authors [11], studying forces on maxillary incisors in patients with partially sectioned upper anterior prostheses to accommodate pressure sensors, found positive and negative data, with some moments when the upper lip tended to pull the labial surface outward. They justified those negative values through the force of gravity acting on the head when inclined forward, and they could not quantify that negative mean value of force. Others [12] studied lip pressures on anterior mandibular areas before and after orthodontic arch expansion to see if they played a role in relapse. They used pressure transducers applied to the vestibular face of mandibular incisors and detected variable pressures during the rest, both negative and positive. Unfortunately, they were unable to achieve a comprehensive explanation; they thought negative values were due to errors in the methodology. Thüer et al. attempted to measure the pressure exerted by cheeks and tongue on the maxillary and mandibular posterior regions in 24 students at rest [13]. They used polyethylene tubes attached to teeth via resin shields and found mainly positive values. Although they found relatively negative pressures at rest on the palatal vault in 50% of the subjects, they also occasionally found negative values even at the vestibular level. Moreover, no correlation between the palatal vault and vestibular area was found, and the authors were unable to provide an explanation for those results.

However, none of these studies aimed to monitor resting intraoral pressure at the gum level. In these experiments, the interest was centered around muscle force, and negative values were sporadic.

**Figure 1:**
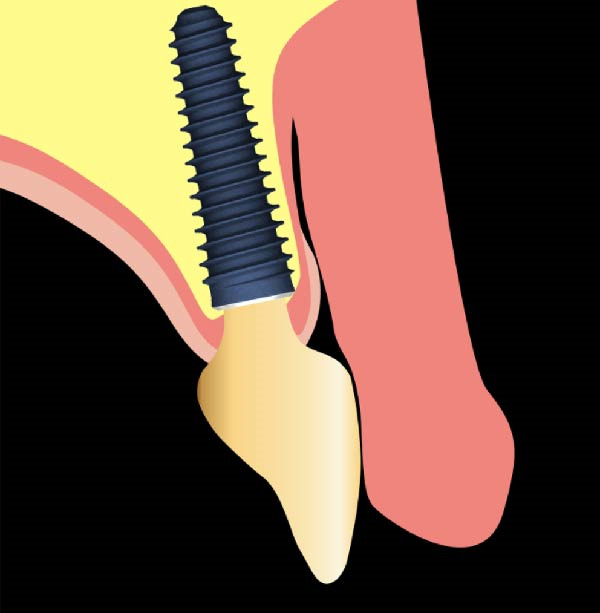
Suction chamber.

In a closed system constituted by soft walls, as the mouth is at the vestibulum, when a negative pressure is applied, it results in pressure between the collapsing walls. Given an instrumental facility to measure the pressure, it failed, between previous studies, to calculate the actual negative pressure that induced the compression effect. In other words, if part of the soft wall is prevented from collapsing, there might be created a “Suction Chamber” [5] where only the negative pressure has an effect (Figure 1). Between the tooth and the gum, a convex buccal outline, at buccal zenith, may prevent the collapse of the cheeks and lips through a mechanical support, avoiding contact with the free gingival margin area, thus obtaining a pulling force that might justify the enigmatic creeping phenomenon described by Agabiti. Hence, quantifying the pressure difference could create a gnoseological tool in order to increase the ability to guide tissue at the optimal contour and increase the control of the long‐term esthetic results around implants and natural teeth. The aim of the article is to find new data about oral pressure to compare with the results found in the studies on swallowing [8–10] previously introduced, as well as with the values collected by studies [11–13] that evaluated intraoral pressure exerted by cheek and lip muscles, but focusing more on the negative values for the possible effects on the gum trophism according to the hypothesis based on Agabiti I [5]. and Rodriguez X [7]. studies. Thus, the findings could possibly support or challenge theories regarding the maturation of soft tissues around implants [5].

## 2. Materials and Methods

To measure the pressure in the oral vestibule, a new instrument has been developed. To avoid measuring the pressure exerted by the muscles on teeth and gums, it is necessary to place a device inside the oral cavity that separates the cheeks and lips from the dental and gingival surfaces. The pressure gauge consists of a constant‐diameter polyethylene tube whose end portion that goes into the mouth is sectioned like a dovetail. This peculiar design was shown, in a previous attempt, to prevent the closure of the terminal by the check collapse, thus altering the measured values.

The device is based on the concept of communicating vessels (S. Stevino, 1568); a standard quantity of water, in small volumes, can be moved by pressure variations generated at the oral level.

The instrument consists of a panel positioned perpendicular to a support plane, on which a sheet of millimeter paper has been applied. On the surface of the panel, the polyethylene tube is glued and shaped to create a loop with two perfectly parallel lines that coincide with the lines of the underlying millimeter paper and are perpendicular to the ground. On one side of the loop, the tube terminates glued to the panel; on the other side, it continues freely to be brought into the oral cavity. Both terminals are not closed.

The loop is partially filled with water colored with drops of methylene blue to make the movements more visible, and the initial level of the water is marked as zero on the millimeter paper; changes in water levels indicate exactly the pressure variations since the diameter of the tube is constant, and the underlying millimeter paper allows these variations to be measured in millimeters of water. The liquid is poured into the tube using a pipette.

All values were recorded in millimeters of water (mmH_2_O) by observing the water level in the tube relative to the underlying millimeter paper. The study included two participants: subject 1 (male) conducted 75% of the total measurements (45 tests), while subject 2 (female) conducted 25% (15 tests). The patients have been involved when the inclusion criteria shown in Table 1 are respected. In this preliminary study, the distribution of the test is not equally covered by the two subjects, and this represents one of the limits of the study. Positions were categorized as awake anterior (AWA) region measurements, awake posterior (AWP) region measurements, and sleep measurements in the posterior region (SLP). It was not possible to measure the pressure in the anterior regions during sleep, since the lip seal was lost in most of the sleep measurements. In contrast, by positioning the tube behind the lip seal, the lip seal was better maintained while still accepting moments when this seal was inevitably lost during sleep.

**Table 1:** Gender and age of the patient have been registered.

Inclusion and exclusion criteria	Subject number 1	Subject number 2
Age	25	24
Gender (male: 1; female: 0)	1	0
Systemic diseases (yes: 1; No: 0)	0	0
Smoke (yes: 1; no: 0)	0	0
Hard or soft‐tissue augmentation (yes: 1; no: 0)	0	0

*Note*: The patients have been included only if systemic diseases, smoking habits, and previous hard or soft‐tissue augmentation procedures are not present. The table’s main aim is to describe patient selection criteria and general characteristics.

**Figure 2:**
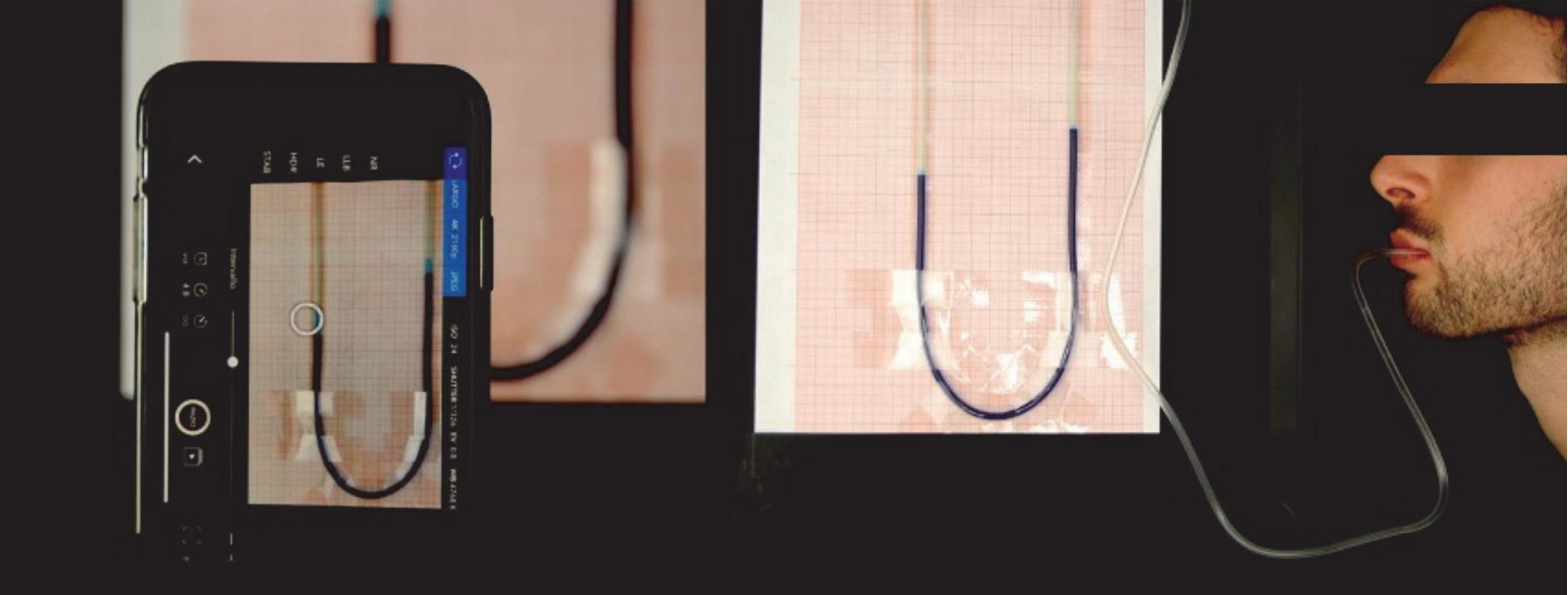
Measurement method.

The pressure values were recorded by taking a sequence of photographs with a smartphone positioned in front of the apparatus as represented in Figure 2. Specifically, the Skyflow application version 1.3.2.3 was used, which allows setting the number of photos and the interval time between each one. At the end of each test, the subject reviews the photos one by one and records the pressure values (determined by the water level on the millimeter paper) on a worksheet paper. In this way, a table is created with all the measurements.

Each test lasted 20 min, with pressure recorded every 5 s. Between an average of 240 measurements, only the final 200 were considered valid to account for acclimatization to the tube in the mouth. The subject was blinded from the results since he could not observe real‐time pressure changes nor the pictures captured.

### 2.1. Data Analysis

Data analysis was conducted using R software version 4.2.1. Descriptive statistics were calculated, including mean, standard deviation, minimum, and maximum for each test.

A purely descriptive statistical approach was adopted due to the limited number of participants (*n* = 2). For each recording condition—AWA, AWP, sleep posterior (SLP), and for the overall dataset—the following metrics were computed: number of observations, mean, median, standard deviation, minimum and maximum values. No inferential analytical techniques (e.g., *t*‐tests or ANOVA) were applied, as these methods are not appropriate for the current sample size.

## 3. Results

A total of 12,000 pressure values has been recorded, and the average value was −17.02 mmH_2_O, which corresponds to −1.25 mm of mercury. The minimum recorded value was −65 mmH_2_O, while the maximum was 20 mmH_2_O.

The mean values appear consistent both between the two subjects and across different measurement modes. For the total pressure values, the mean for subject 1 was −17.05 mmH_2_O, while for subject 2 it was −16.92 mmH_2_O. For the total measurements, the standard deviation for subject 1 was 5.89, while for subject 2 it was 13.66.

### 3.1. Descriptive Statistics

A total of ~12,000 valid pressure recordings were analyzed across 60 tests (45 males; 15 females). Values represent pressure variations inside the oral vestibule during resting conditions, captured at 5‐s intervals. Descriptive statistics of vestibular pressure across conditions indicated high consistency between the different measurement modes. Mean pressure values remained within a narrow range between 16 and 18 mmH_2_O, with comparable median levels (Table 1). Minor variability was observed in sleep posterior conditions (SLP), where sporadic lip‐seal loss produced isolated positive peaks up to 250 mmH_2_O, though these episodes did not influence the overall resting trend.

**Figure 3:**
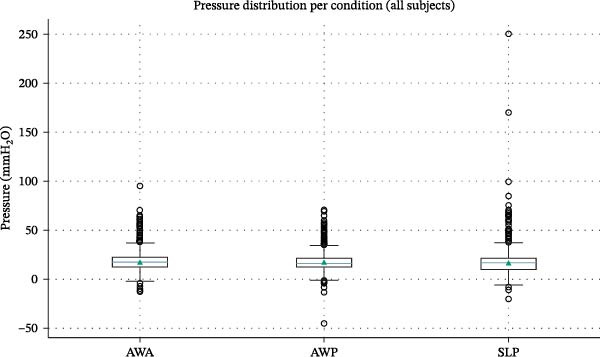
The graphic represents the distribution of pressure measured in different conditions (AWA, awake anterior region; AWP, awake posterior region; SLP, sleep posterior region) calculated in mmH_2_O.

Figure 3 illustrates the distribution of pressure values for AWA, AWP, and SLP conditions using box‐and‐whisker plots. AWA showed the highest mean and narrow dispersion, suggesting that the anterior region during wakefulness maintains a stable sub‐atmospheric environment. AWP and SLP displayed greater intracondition variability, consistent with expected functional fluctuations in the posterior vestibule, particularly during sleep when lip seal is intermittently compromised. Importantly, median values across all three conditions remain closely aligned, reinforcing the presence of a uniform residual negative pressure state in the vestibular compartment.

**Figure 4:**
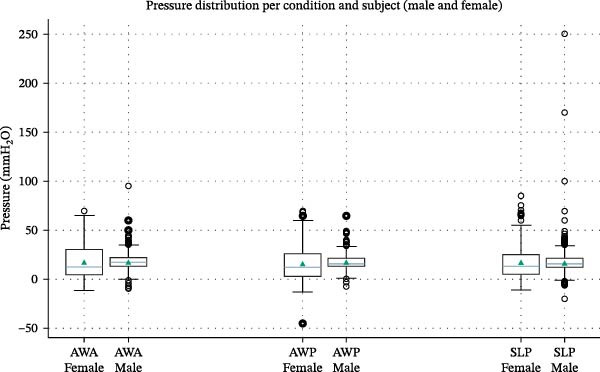
The graphic represents intraoral pressure in different conditions, distinguishing the two different subjects (for each condition, on the left the female measurement and on the right the male one), again measured in mmH_2_O.

Figure 4 shows the same boxplot representation stratified by subject (male vs. female). Both individuals demonstrated comparable median values and overlapping variability ranges. The male subject showed more extreme values (both negative and positive), likely due to a broader range of vestibular adaptability or slight behavior changes while maintaining lip seal during extended recording periods. Despite this, the central tendency is strikingly similar between subjects, supporting the hypothesis that resting vestibular pressure is not a highly individual‐dependent parameter in physiological conditions.

**Figure 5:**
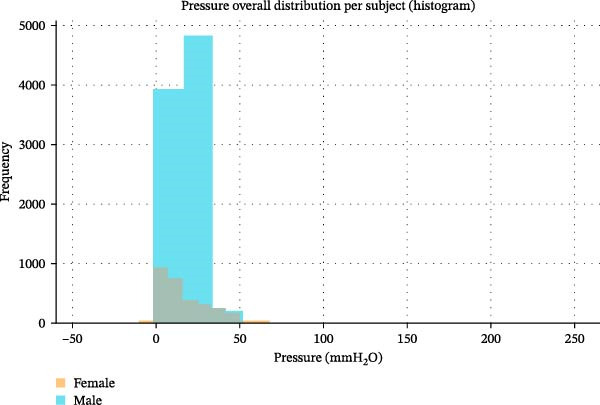
The graphic represents pressure values revealing frequency distribution. In blue the values referred to the male subject; in yellow the results referred to the female one.

Figure 5 depicts histograms of all pressure measurements per subject, revealing the frequency distribution of the recorded pressures. Both subjects show a pronounced clustering of values around 10–25 mmH_2_O, while negative values (i.e., under‐pressure conditions) and high positive peaks appear as physiologic outliers only. This may confirm a predominantly sub‐atmospheric vestibular pressure regime and infrequent deviations from resting homeostasis. The bimodal shoulder in the male distribution reflects transient lip‐seal recovery cycles, inherently associated with maintaining oral negative pressure.

**Figure 6:**
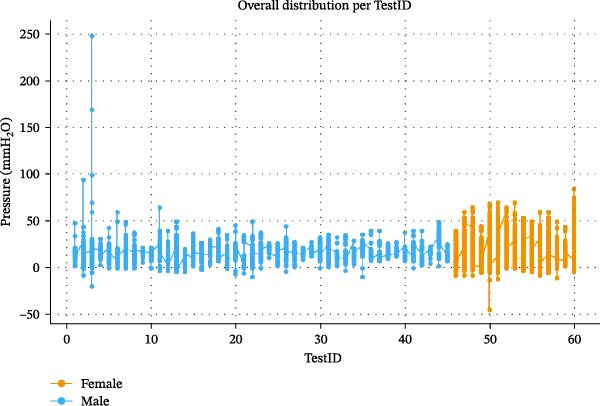
The graphic shows the values distribution based on subject (male: blue; female: yellow) at different TestIDs from the first to the sixtieth.

Figure 6 presents the temporal evolution of pressure values across test sessions (TestID), separated by subject. The traces exhibit a highly stable pattern around the mean, with typical micro‐oscillations linked to natural micromovements of lips and cheeks at rest. No progressive drift, fatigue pattern, or adaptation effect was observed throughout the 20‐min tests, showing a repeatable trend of the methodological setup. Morover the vestibular pressure condition seems to be stable, and in the end, there is an absence of temporal bias in liquid‐level readings.

Across all figures and descriptive statistics, the results tend to substantiate that resting vestibular pressure is consistently slightly negative compared to atmospheric pressure. In addition to that, variability between subjects and sites is minimal and physiologically explained. Finally, it seems that negative pressure values represent a stable and reproducible physiologic state, not an episodic artifact.

This supports the interpretation of a residual intraoral “suction chamber effect” during rest.

## 4. Discussion

The descriptive observations reinforce the hypothesis that a stable residual negative pressure is present in the oral vestibule during rest conditions. The trends appear comparable across recording sites and physiological states (awake vs. sleep), suggesting that this pressure equilibrium may represent a baseline functional condition of the oral cavity rather than a dynamic phenomenon restricted to swallowing phases. Although variability exists—particularly in male subject measurements—the overall range remains physiologically coherent, supporting the conceptual model of a suction chamber, potentially influencing gingival trophism through mechanotransduction and microcirculatory modulation.

About the issue of lip seal during sleep, despite the tube being positioned in the posterior region, this seal was unavoidably lost on some occasions. However, the values recorded during those instants were still considered valid since the main objective of this study was to measure the average resting pressure, therefore including moments of spontaneous lip‐seal loss.

The most significant finding of this study is the average resting pressure at the oral vestibule level of −1.25 mmHg. This aligns with studies on swallowing [8–10], suggesting that a slight negative pressure remains post‐swallowing, although much lower than during the act itself. Several mechanical explanations exist for the resting relative negative pressure in the oral vestibule. First, it promotes a lip seal, preventing saliva leakage. Additionally, oral vacuum causes soft tissues to occupy most spaces, possibly reducing saliva consumption. It also may reduce the work for the mandible elevator muscles to maintain position against gravity. The pressures recorded fit with previous studies that evaluated intraoral pressure exerted by cheek and lip muscles [11–13], but, additionally to the findings of these authors, the focus was directed to the negative values. The simultaneous presence of both negative and positive pressure values could be due to the fact that where there is contact between the surfaces of muscles, alveolar mucosa, gum, or teeth, there is a positive relative pressure, while in spaces where the mucosa cannot collapse, the pressure is negative. This scenario is common in the esthetic interface between the apical third of the facial crown and the surrounding tissues, as well as in the papilla zones.

This finding of −1.25 mmHg supports Agabiti’s theory on peri‐implant tissue maturation. Negative gradient of pressure, through suction effect, might drive the growth of the gingival margin and interproximal papilla [5]. However, this growth can only occur if all biological principles regarding implant placement and site anatomy are respected, including having adequate vestibular bone [14], implant platform distance from the mucosal surface [15], a band of keratinized mucosa [16], compliance with Nozawa’s parameters [17], an ideal implant design [18] with an efficient implant‐abutment connection [19], and specific implant emergence profiles [1]. Especially on implants, this effect may be obtained without the passive orthodontic migration that affects the natural teeth; in this biological model, the facial convexity of the prosthetic element may assume the role of soft‐tissue support, thus designing a suction chamber at the buccal crown‐to‐tissue interface (Figure 1).

The relationship between tissue volumetric response and negative pressure gradient could be biologically motivated through the mechanotransduction mechanism [20, 21]. The development, maintenance, and remodeling of connective tissue rely on physical load [22], with integrin receptors connecting the extracellular matrix to the fibroblast cytoskeleton and transmitting forces bidirectionally. External stress causes matrix deformation, transmitted to the cell’s cytoskeleton; fibroblasts counteract applied force with cytoskeletal traction to maintain shape [20]. “Mechanosensors,” such as adapter proteins and integrin‐associated cationic channels, undergo conformational changes in response to applied force, converting a mechanical signal into a chemical one [21]. This explains why stress from oral vacuum and myofibroblasts’ contraction leads to gingival connective tissue growth [7].

Additionally, the oral vestibule’s negative pressure might influence gingival microcirculation. The gingival tissue is fed by capillaries, which are the sites of exchange between blood and the interstitial fluid in which the cells of the connective and epithelial tissue are immersed. The dynamics of microvascular fluids are regulated by a revised Starling’s law [23] (Equation (1)), and it establishes that the net flow of water through the endothelial wall (*J*_v_) is given by an equation that depends on the hydraulic conductivity of the vascular barrier (*L*_p_), the difference in hydraulic pressure between the vascular lumen and the interstitial space (*P*_i_), the difference in oncotic pressure between the vascular lumen and the interstitial space (Δ*π*), and the protein reflection coefficient of the endothelial wall (*σ*).

(1) Jv=Lp⋅ S⋅ΔPi−σΔπ.

The negative relative pressure recorded inside the oral cavity could reasonably be added to the interstitial hydraulic pressure, comparably to what happens at the transmural level in the subpleural space during ventilation [24]. This would favor a net positive pressure gradient and thus greater outflow of water and solutes, resulting in an improvement in nutrients for the nearby cells and thus an advantage on the trophism of the gingival tissue.

At the capillary level, there are two opposing forces that regulate microcirculation: filtration pressure, which causes the outflow of fluid from the vessel and results from the sum of capillary hydraulic pressure, interstitial oncotic pressure, and interstitial hydraulic pressure when negative. Reabsorption pressure, which promotes the reabsorption of fluid from the interstitium into the vessel and the lymphatic system, is constituted by capillary oncotic pressure and interstitial hydraulic pressure when more positive than the hydraulic pressure of the capillary lumen.

Under normal conditions, the filtration pressure at the arterial end of the capillary vessel has a value of about 36 mmHg, while it drops to about 21 mmHg at the venous end. The reabsorption pressure is about 28 mmHg [25]. This results in a net filtration flow sustained by a net pressure gradient of 8 mmHg at the arterial end and a net reabsorption flow sustained by a net pressure gradient of 7 mmHg at the venous end.

In the oral cavity, negative interstitial hydraulic pressure of 1.25 mmHg should be added to the filtration pressure, leading to a net filtration of 9.25 mmHg instead of 8 mmHg at the arterial end. At the venous end, the net reabsorption flow reduces from 7 mmHg to 5.75 mmHg (Figure 7).

Figure 7(a) Fluid dynamics on microvascular circulation. (b) Effect of relative intraoral negative pressure on microvascular circulation.

**Figure:**
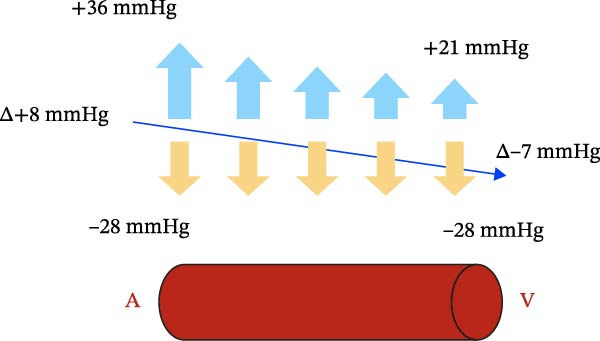
(a)

**Figure:**
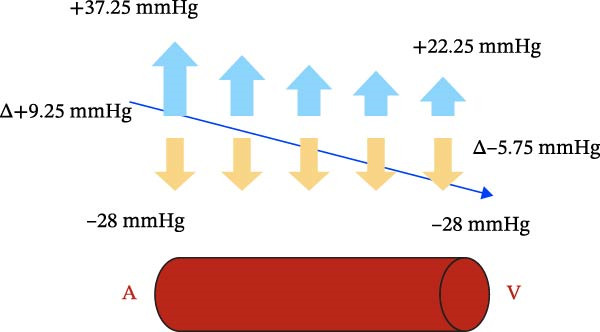
(b)

Authors should discuss the results and how they can be interpreted from the perspective of previous studies and of the working hypotheses. The findings and their implications should be discussed in the broadest context possible. Future research directions may also be highlighted.

### 4.1. Limitations of the Present Study and Possible Research Projects

**Figure 8:**
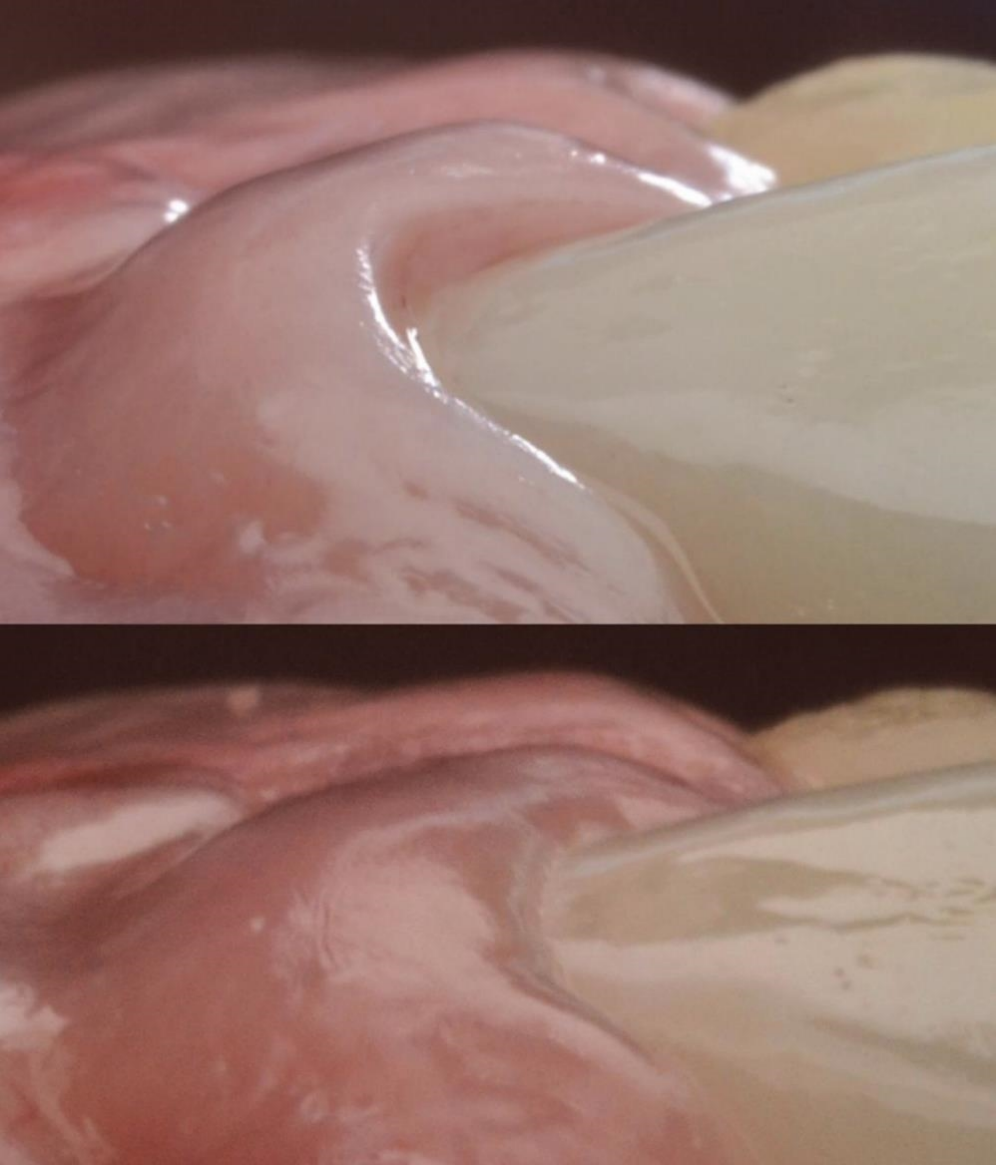
Soft tissue maturation in an implant‐supported prosthesis.

The primary limitation of this investigation is the restricted sample size and dataset consisting of only two subjects, thus preventing formal inferential conclusions. In addition, occasional lip‐seal loss during sleep recordings may have contributed to isolated high outliers; nonetheless, these did not alter the stability of the mean resting values. Future studies should expand the population and incorporate real‐time continuous monitoring systems, enabling mixed modeling approaches to evaluate inter‐ and intrasubject variability more accurately. Additional clinical trials should further investigate the potential role of vestibular negative pressure as a mechanobiological stimulus for soft‐tissue maturation around natural teeth and implants (Figure 8).

## 5. Conclusions

The present findings indicate the presence of a stable residual negative pressure within the oral vestibule during resting conditions, consistently observed across subjects, test locations, and wakefulness or sleep states. This suggests that a physiologic vacuum state may represent a baseline condition of the oral cavity, possibly contributing to soft‐tissue trophism through mechanotransduction mechanisms and microcirculatory modulation.

Although these observations align with the proposed suction chamber concept and connect well with the clinical evidence regarding peri‐implant mucosal maturation, further investigation is required to validate these biological implications. Larger controlled studies, including advanced quantitative methodologies, are necessary to confirm the clinical relevance and therapeutic potential of resting intraoral negative pressure in guiding soft‐tissue behavior around natural teeth and implants.

## Author Contributions

Conceptualization: Davide Farronato. Methodology: Davide Farronato, Gabriele Dani, and Piero Antonio Zecca. Validation: Andrea Moriondo and Davide Farronato. Formal analysis: Leonardo Romano and Lorenzo Azzi. Investigation: Gabriele Dani. Data curation: Gabriele Dani and Piero Antonio Zecca. Writing – original draft preparation: Dani Gabriele and Piero Antonio Zecca. Writing – review and editing: Davide Farronato, Andrea Moriondo, and Gabriele Dani. Supervision: Lorenzo Azzi. Project administration: Davide Farronato.

## Funding

The authors received no specific funding for this work. Open access publishing facilitated by Universita degli Studi dell′Insubria, as part of the Wiley ‐ CRUI‐CARE agreement.

## Disclosure

All authors have read and agreed to the published version of the manuscript.

## Ethics Statement

The study was approved by the Ethics Committee of the Ospedale di Circolo e Fondazione Macchi in Varese (Approval Number 826 03/10/2013). All participants provided informed consent in accordance with the Declaration of Helsinki.

## Consent

Informed consent was obtained from the subject involved in the study. Written informed consent has been obtained from the patient to publish this paper.

## Conflicts of Interest

The authors declare no conflicts of interest.

## Data Availability

The data presented in this study are available upon request from the corresponding author.
